# The Impact of Dexamphetamine Treatment for Obesity on Executive Function: A Double-Blind Randomised Controlled Pilot Study

**DOI:** 10.3390/brainsci14121274

**Published:** 2024-12-18

**Authors:** Antoinette Poulton, Natalie Gauci, Hazer Khalifa, Emily J. Hibbert, Alison S. Poulton

**Affiliations:** 1 Melbourne School of Psychological Sciences, University of Melbourne, Parkville, VIC 3052, Australia; 2 Nepean Clinical School, Faculty of Medicine and Health, University of Sydney, Sydney, NSW 2751, Australia; natalie.gauci@nepeanlungandsleep.com.au (N.G.); hazer.khalifa@sydney.edu.au (H.K.); emily.hibbert@sydney.edu.au (E.J.H.); alison.poulton@sydney.edu.au (A.S.P.); 3 Charles Perkins Centre-Nepean, Faculty of Medicine and Health, University of Sydney, Sydney, NSW 2003, Australia; 4 Nepean Hospital, Penrith, NSW 2747, Australia

**Keywords:** obesity, dexamphetamine, executive function, response inhibition, attention-deficit/hyperactivity disorder, substance use disorder

## Abstract

Background: Amphetamines increase dopamine levels in mid-brain regions which, in turn, impact top-down executive function. Repeated exposure is linked to substance use disorders. Nonetheless, amphetamines are used to manage attention-deficit/hyperactivity disorder (ADHD) and eating-related disorders. In ADHD, amphetamines upregulate a system characterised by low dopaminergic tone, assisting to improve executive function. A similar process might be at play with eating disorders; however, the effect of amphetamine treatment on executive function in this case has not been thoroughly considered. Methods: Participants (*N* = 52, *M_age_* = 47.06, *SD* = 12.29) with a body mass index of 25–60 were randomised to treatment (6-week dexamphetamine titration) or control (placebo) groups. They completed an executive function measure—Barkley Deficits in Executive Functioning Scale (BDEFS-SF)—and response inhibition task—Stop-Signal Task (SST)—at Baseline, throughout titration, at Maintenance, and at Follow-up. Mixed effects models examined whether BDEFS-SF score or the SST variable, stop-signal reaction time (SSRT), changed across sessions as a function of treatment. Results: There was no effect of group (*p* = 0.440), but an effect of session (*p* = 0.024) on BDEFS-SF, with scores at Time 2 (*p* = 0.011, 95% CI [0.47, 3.49]) and Maintenance (*p* = 0.022, 95% CI [−4.89, −0.39]), respectively, higher and lower than other timepoints. There was no group by session interaction (*p* = 0.659). *R*^2^ (conditional) = 0.74; ICC = 0.73. There was an effect of group (*p* = 0.039) and session (*p* < 0.001) on SSRT, but no interaction (*p* = 0.707). Baseline SSRT was significantly longer than the mean of all subsequent timepoints (*p* < 0.001, 95% CI [16.29, 33.84]). *R*^2^ (conditional) = 0.47; ICC = 0.39. Conclusions: There was no discernible impact of amphetamine treatment for obesity on executive function. Our results suggest some variation related to sample size and/or practice effects. Thus, while treatment appears unlikely to render individuals susceptible to substance use disorders, parallels with ADHD might be overstated.

## 1. Introduction

Amphetamines (alpha-methylphenethylamine) refer to a diverse category of drug that include synthetic compounds, such as dexamphetamine (DEX), methamphetamine (METH), methylenedioxymethamhetamine (MDMA), cocaine, and methylphenidate, as well as naturally occurring ones, like ephedrine and cathinone [1,2,3]. Although these psychostimulants are often loosely grouped together in the literature [4], there are subtle differences related to the structure and mechanisms of effect [1,5], with several endorsed in the treatment of some medical conditions. DEX, methylphenidate, and lisdexamfetamine are routinely prescribed for attention-deficit/hyperactivity disorder (ADHD), for instance, while lisdexamfetamine has more recently also been used in the treatment of binge eating disorder [6,7,8]. Nonetheless, apprehension related to the addictive properties of these drugs—even when prescribed by a medical practitioner—has often been articulated [2,9,10]. This has potentially led to a reluctance to further examine the therapeutic potential of some of these drugs. Charting the impact of amphetamines with therapeutic potential in areas of cognition implicated in models of addiction might assist to alleviate concern and may lead to a more nuanced approach when considering future pharmacological possibilities.

Concerns related to the addictive properties of amphetamines centre on the role of dopamine (DA) in the development and maintenance of substance use disorders (SUDs). Increased concentrations of DA induced in midbrain regions by amphetamines (and a wide variety of other illicit substances) are immediately rewarding, and thus, positively reinforcing [11,12]. This drives adaptive behaviour, with actions leading to reward likely to be repeated [11]. In turn, this produces a conditioned response such that increases in DA occur in response to cues (paraphernalia, emotion, etc.) that predict drug taking. With further intake, the conditioned memory is reinforced, and the cues begin to prompt the addictive behaviour [12]. Critically, this disruption to DA-mediated mid-brain circuitry also impacts top-down executive function mechanisms, particularly in regions that underpin the ability to successfully withhold dominant behavioural responses, or response inhibition [11]. Several prominent, conceptually similar models of addiction suggest that the following imbalance emerges: there is simultaneously heightened impulsivity for the rewards associated with taking drugs (pleasure of intoxication and alleviation of craving) and a diminished capacity to control this impulsivity [11,13,14,15,16,17,18]. Indeed, a large body of evidence shows persons with clinically diagnosed SUDs, including those involving amphetamines, are characterised by increased impulsiveness for reward and/or impaired executive function [11,19,20]. The sub-clinical and recreational use of amphetamines has also been linked to executive dysfunction [21].

Nevertheless, amphetamines are routinely utilised in the management of ADHD and are, increasingly, being considered for the treatment of eating-related disorders. In the case of ADHD, prescribed stimulants are thought to upregulate a system characterised by genetically mediated low dopaminergic tone [2,22]. Imaging studies suggest individuals with ADHD have low DA receptor expression, as compared to healthy controls, meaning midbrain DA neurotransmission is attenuated [23,24]. This is proposed to give rise to the behavioural manifestations—related primarily to executive dysfunction—of the disorder [25,26]. Amphetamines enhance neurotransmission, assisting to normalise DA levels in the brains of individuals with ADHD [22]. In turn, this stabilises behaviour such that improvements in executive function are evident [21,27,28,29]. A similar process is thought to be at play in the case of eating-related disorders. Generally, there is high comorbidity between ADHD and eating disorders, and emerging findings suggest substantial neurobiological overlap [6,26,30,31]. Low dopaminergic tone has been linked to food restriction, binge eating behaviours, and obesity [6,26,32,33,34]. There is also some evidence of associated impulsivity and executive dysfunction, including difficulties with response inhibition [35,36,37]. A systematic review found that participants with obesity performed significantly worse on various objective measures of response inhibition, including the Stop-Signal Task (SST), as compared to healthy controls, and that these individuals were characterised by decreased prefrontal brain activation during tasks [36]. While amphetamine-based treatments have shown promise in terms of reducing symptomatology in the case of binge eating disorder [7,8,38], there has been very limited examination of the impact of this treatment on cognition. Two studies have together shown that impulsivity, as measured by a self-report survey, is significantly reduced following lisdexamfetamine treatment for binge eating disorder, suggesting improved executive function in the treatment group [32,39]. However, no change was evident on a cognitive measure of response inhibition [39]. Regardless, further research is warranted. Questions remain regarding whether improvements in executive function, including response inhibition, might be evident when amphetamine treatment is applied to other eating-related disorders and whether there is no negative impact on executive function that might, in turn, render these individuals vulnerable for the development of SUD.

Using a double-blind randomised controlled pilot design, we sought to consider the cognitive impact of using DEX to treat obesity in otherwise healthy individuals. We aimed to investigate whether executive function generally or response inhibition specifically changed over time in response to treatment. Any evidence of diminished executive function or reduced response inhibition could suggest SUD susceptibility. Considering the literature, however, it was hypothesised that, compared to controls, participants treated with DEX would exhibit enhanced executive function—as measured by a self-report survey—and improved response inhibition—as assessed via the SST—over time in response to DEX titration. Following the treatment phase, executive function and response inhibition were expected to return to baseline levels.

## 2. Materials and Methods

### 2.1. Participants

**Figure 1:**
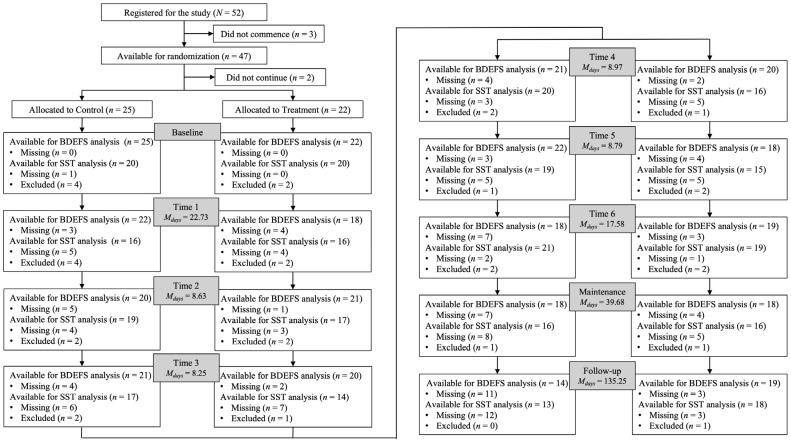
CONSORT diagram.

Fifty-two individuals (*M_age_* = 47.06, *SD* = 12.29, range: 23–68; 63.5% male) were recruited from outpatient clinics for a double-blind randomised controlled trial investigating the efficacy of DEX as a treatment for obesity complicated by obstructive sleep apnoea (OSA; Registration number: ACTRN12614000654651; Universal trial number: U1111-1157-8317). Adults aged 18–70 years with a body mass index of 25–60 that met the criteria for nocturnal respiratory support for OSA were eligible for inclusion. Participants were excluded if they had a history of illicit drug addiction; uncontrolled hypertension; heart, kidney, or liver disease; uncontrolled epilepsy; were breastfeeding, pregnant, or planning for a pregnancy; had a history of bariatric surgery; had a current diagnosis of depression or other psychiatric illness; were currently/recently being treated with psychotropic medication, systemic glucocorticoids, or weight loss medications; had a family history of sudden death from cardiac causes; hypersensitivity to DEX or any components of the DEX or placebo tablet; or if they did not continue to baseline testing. After exclusions, data from 47 individuals were available for analysis (*M_age_* = 47.72, *SD* = 12.16, range: 23–68; 68.09% male). Further participants were excluded at each timepoint due to missing self-report survey data or as a function of cognitive task compliance criteria. See Figure 1 for CONSORT diagram. The study was approved by the Nepean Blue Mountains Local Health District Human Research Ethics Committee (2019/ETH01361) and was conducted in accordance with the National Health and Medical Research Council standards for ethical research. Participants provided informed consent.

### 2.2. Procedure

At enrolment, participants undertook baseline medical assessments and completed demographic and other surveys pertinent to the weight loss/OSA component of the study. They also completed the Barkley Deficits in Executive Functioning Scale—Short Form (BDEFS-SF) and the SST, a cognitive task designed to assess response inhibition. Participants were then randomised into the treatment (*n* = 22) or control (*n* = 25) group by pharmacists at the Nepean Hospital (Penrith). Randomisation was 1:1 using a block size of 4. Those in the treatment group were subject to DEX titration. They commenced by taking 5 mg tablets twice daily (10 mg total) followed by weekly incremental doses of 5 mg twice daily to a maximum of 30 mg twice daily (60 mg total). Participants continued taking the maximum dose over the remainder of the 6-month treatment period (Maintenance), except in the last month when they were back-titrated off the medication. Where treatment group participants received DEX, controls received placebo medication. The DEX and placebo tablets and packaging were identical. All participants were required to complete the BDEFS-SF and SST each week during the titration phase (Times 1–6) as well as at least once during both the Maintenance and Follow-up periods. It should be noted that the COVID-19 pandemic and associated lockdowns impacted protocol timelines and thus hampered data collection. In Australia, different regions experienced lockdowns and/or restrictions at different moments in time depending on the COVID-19 caseload in that area. As such, some persons would have been free to attend the laboratory for testing while others would not have been allowed to leave their home. Additionally, during this time, persons diagnosed with COVID-19 were required to isolate for 14 days, and there were periods when the hospital where this research was conducted was closed to all visitors. Figure 1 shows how the timelines were impacted and the extent of missing data.

### 2.3. Measures

#### 2.3.1. Barkley Deficits in Executive Functioning Scale—Short Form (BDEFS-SF)

Derived from the Barkley Deficits in Executive Function—Long Form, the BDEFS-SF comprises 20 self-report items assessed on a 4-point Likert scale that assess self-organisation/problem solving, self-management of time, self-restraint, self-regulation of emotion, and self-motivation [40,41]. Characterised by very high internal consistency (α = 0.92) and good test–retest reliability (0.62–0.80), the BDEFS-SF is considered psychometrically sound [40]. Scores range 20–80; higher scores indicate greater executive dysfunction.

#### 2.3.2. Stop-Signal Task (SST)

The SST was programmed using Inquisit 4.0.10.0 Lab [42] and delivered in the laboratory via a dedicated MacBook Pro with a 13-inch monitor. Widely used to assess response inhibition in neuroscience, psychiatry, and psychology [43], the task consists of a practice block of 32 trials and three blocks of 64 experimental trials. Each trial commenced with a white fixation cross (500 ms) followed by the presentation of a go stimulus (an arrow within a circle pointing either left or right). Participants were required to press the corresponding arrow on the keyboard as quickly as possible. In 25% of trials, an auditory beep followed the presentation of the go stimulus, signalling a stop trial. Stop signals were presented at random and not in consecutive trials. Participants were required to withhold their response in stop trials. The initial stop signal delay (SSD) was set at 250 ms and adjusted dynamically as a function of participant response; successful inhibitions resulted in a 50 ms increase in the SSD (to a maximum of 1150 ms), while unsuccessful inhibitions decreased it by 50 ms (to a minimum of 50 ms). Go RT is considered an indicator of sustained attention [44]. Stop-signal reaction time (SSRT) was derived when mean SSD was subtracted from average go RT; greater SSRTs indicate reduced response inhibition [45,46]. Participants were excluded if stop accuracy was less than 25% or greater than 75%; go accuracy was less than 60%; go errors were greater than 10%; or if SSRT was less than 50 ms [43,47,48]. The full task took approximately 12 min to complete.

### 2.4. Data Analyses

Independent *t*-tests and Chi-square analyses were conducted to determine whether there were any demographic differences as a function of group at baseline. Effect sizes were computed for *t*-tests using Cohen *d* and were interpreted in accordance with Cohen guidelines: small effect = 0.20, medium effect = 0.50, and large effect = 0.80; for Chi-square, Cramer’s *V* was utilised as a measure of effect: 0.07 = small, 0.21 = medium, and 0.35 = large effect [49]. Repeated measures mixed ANOVA was utilised to investigate changes in weight across time as a function of treatment group, with time (Baseline, Follow-up) as the within-subjects factor and group (treatment, control) as the between-subjects factor. Effect size was calculated using *ω*^2^: 0.01 = small, 0.06 = moderate, and 0.14 = large effect [50]. Post hoc tests were Bonferroni-corrected for multiple comparisons. Correlations were used to determine whether there was any relationship between the dependent variables of interest and participant age.

Mixed effects models were computed using Jamovi (2.3.28.0) to examine whether the BDEFS-SF score or SSRT differed across sessions or as a function of treatment group. The BDEFS-SF model comprised 279 observations, while the SST model contained 312. Mixed effects models are recommended where multiple—especially longitudinal—measurements are taken from participants and where there might be missing values and uneven spacing of measurements [51]. Unlike other models which discard data from a participant when a single measurement is missing, mixed effects models allow other data from participants to be utilised so long as the missing data are missing-at-random. Additionally, multiple measures taken from the same participants are likely to be correlated in various ways and this violates assumptions related to independence [51]. Mixed models account for these correlations [52].

Session (Baseline, Times 1–6, Maintenance, Follow-up) and treatment group (control, treatment) constituted fixed effects, while participant ID was included as a random effect. Models were computed using both maximum likelihood estimation and restricted maximum likelihood estimation (REML) methods. Akaike information criteria, Bayesian information criterion, and Log likelihood fit statistics of converged models were examined, and the model with the best fit—represented by lower absolute values—was adopted [53]. In each model, the REML method provided the best fitting models. Residuals were checked for normality via the inspection of Q-Q plots. Type III tests were used as these are recommended when data are considered unbalanced. The Wald interval was utilised to determine confidence intervals, while the Satterthwaite method was used to compute degrees of freedom for fixed effects *p*-values [53]. Model equation is determined as follows:

Outcome ~ 1 + Session + Treatment + Session:Treatment + (1|ID)

As advocated, marginal and conditional *R*^2^ indices are reported [54]. Intraclass correlation (ICC) indices are also reported; values of 0.50–0.75 indicate moderate reliability [55]. Time (in days) was included as a covariate in all models; as this did not substantially alter findings, it was omitted from the final models.

## 3. Results

Independent *t*-tests revealed that treatment (*M_age_* = 44.96, *SD* = 13.64, range: 23–68; 72.7% male) and control (*M_age_* = 50.16, *SD* = 10.37, range: 29–65; 64% male) group participants did not differ significantly on age, *t*(45) = 1.48, *p* = 0.145, *d* = 0.43, weight, *t*(45) = 0.21, *p* = 0.833, *d* = 0.06, or biological sex, *χ*^2^(1, *N* = 47) = 0.41, *p* = 0.522, *V* = 0.09, at Baseline. While these preliminary analyses revealed no statistical differences between the groups, there was nonetheless a medium effect of age. Age was not, however, correlated with our dependent variables of interest—Baseline BDEFS-SF (*p* = 0.155) or Baseline SSRT (*p* = 0.465)—and so was not included as a covariate in inferential analyses. The treatment group reduced weight to a significantly greater extent than the control group from Baseline to Follow-up, *F*(1, 45) = 160.06, *p* < 0.001, *ω*^2^ = 0.03.

Table 1 shows mean BDEFS-SF scores for the whole sample across sessions. There was no correlation between age and Baseline, Times 1 to 6, or Maintenance BDEFS-SF score; there was a significant correlation between age and Follow-up (*p* = 0.019) BDEFS-SF score, with older individuals characterised by lower scores. Table 2 shows average performance on SST variables for the whole sample across sessions after exclusion criteria were applied. There was no correlation between age and Baseline, Times 1 to 6, Maintenance, or Follow-up SSRT. Average minutes between treatment (DEX or placebo) and undertaking SST testing is shown in Table 3.

**Figure 2:**
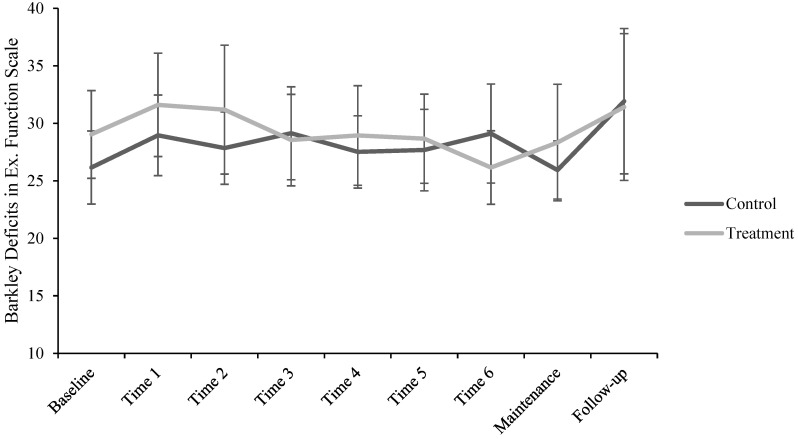
Barkley Deficits in Executive Functioning Scale—Short Form scores across sessions as a function of treatment group.

For the BDEFS-SF model, fixed effects omnibus tests revealed no effect of group on mean score, *F*(1, 45.32) = 0.61, 95% CI [−2.35, 5.46], *p* = 0.440. There was an effect of session, *F*(8, 234.82) = 2.26, *p* = 0.024, but no group by session interaction, *F*(8, 234.82) = 0.74, *p* = 0.659. Examining fixed effects parameter estimates using helmert coding showed that Time 2 mean BDEFS-SF score was significantly greater than the mean score of all subsequent sessions, *beta* = 1.98 (*SE* = 0.77), *t*(234.31) = 2.57, 95% CI [0.47, 3.49], *p* = 0.011. Mean Maintenance BDEFS-SF score was significantly less than that of Follow-up, *beta* = −2.64 (*SE* = 1.15), *t*(235.41) = −2.30, 95% CI [−4.89, −0.39], *p* = 0.022. No other fixed effects parameter estimates were significant. *R*^2^ (marginal) was 0.04; *R*^2^ (conditional) was 0.74. The ICC value was 0.73. Figure 2 shows the BDEFS-SF scores across sessions as a function of group.

**Figure 3:**
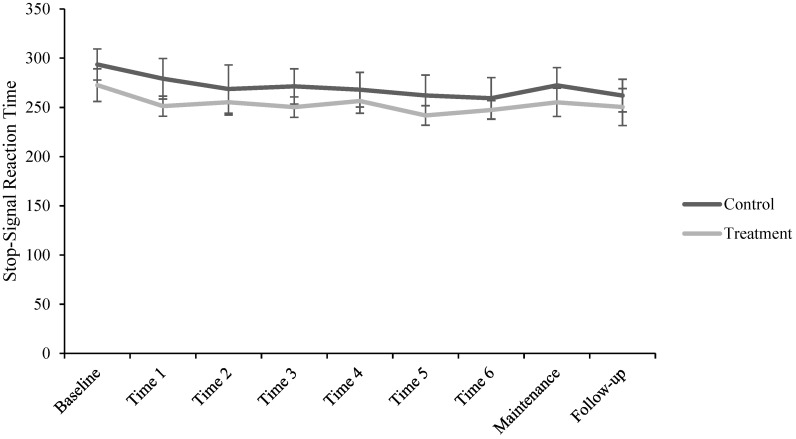
Stop-signal reaction time across sessions as a function of treatment group.

For the SSRT model, fixed effects omnibus tests revealed an effect of group on SSRT, *F*(1, 44.39) = 4.52, 95% CI [−28.26, −1.14], *p* = 0.039, and an effect of session, *F*(8, 255.74) = 5.17, *p* < 0.001. There was no group by session interaction, *F*(8, 255.74) = 0.68, *p* = 0.707. Examining fixed effects parameter estimates using helmert coding showed that mean Baseline SSRT was significantly longer than the mean of all subsequent timepoints, *beta* = 25.06 (*SE* = 4.48), *t*(255.35) = 5.60, 95% CI [16.29, 33.84], *p* < 0.001. Fixed effects parameter estimates using simple coding showed a difference between the treatment and control group on SSRT, *beta* = −14.70 (*SE* = 6.92), *t*(44.39) = −2.12, 95% CI [−28.26, −1.14], *p* = 0.039. No other fixed effects parameter estimates were significant. *R*^2^ (marginal) was 0.12; *R*^2^ (conditional) was 0.47. The ICC value was 0.39. Figure 3 shows SSRT values across sessions as a function of group. See Supplementary Materials for the SST go RT model.

## 4. Discussion

This double-bind randomised controlled pilot study aimed to investigate whether changes in executive function, including response inhibition, were evident in those treated for obesity with DEX. Participants were assessed at Baseline, weekly across a 6-week titration period (DEX or placebo), during Maintenance and at Follow-up. Across Times 1–6 (titration) and at Maintenance, testing occurred at least 2.5 h (but not more than 3.2 h) following ingestion of medication. The pharmacological effects of DEX lasted 4–6 h, with onset of action not greater than 30 to 45 min [56]. As such, we can be reasonably confident participants were experiencing the therapeutic benefits of DEX during each testing session. Nonetheless, contrary to expectations, participants in the treatment group did not report enhanced subjective executive function—as measured by the BDEFS-SF—while taking medication, relative to those in the control group. There was also no evidence of improved response inhibition—assessed via the objective SST—among those in the treatment versus control group. Regardless, patients in the treatment group experienced significant weight loss across the trial.

Mixed effects modelling revealed that, across sessions, there were some minor variations in BDEFS-SF score for the whole sample. There was no main effect of group and, critically, no group by session interaction, suggesting that any changes in score across time were not related to DEX treatment. Our findings do not completely align with several studies that have shown a reduction in self-reported impulsivity—assessed using the Barratt impulsiveness scale (BIS)—in patients being treated with lisdexamfetamine for binge eating disorder [32,39]. While the BIS is regularly employed to demonstrate heightened impulsivity in the context of drug use, SUD, and ADHD [20,57,58,59], emerging evidence also shows that, relative to healthy controls, patients with binge eating disorder are characterised by elevated BIS scores [37]. Improvements in the BIS scores of patients with binge eating disorders during amphetamine-based treatment suggests an enhanced ability to upregulate executive function mechanisms to control heightened impulsivity and contributes to arguments proposing a similar mechanism of effect—related to low dopaminergic tone—between ADHD and eating-related disorders [6].

There could be several reasons why the BDEFS-SF did not show any change in the executive function of our treatment group participants. Derived from a longer 89-item version, the measure is thought to assess cognitive and behavioural indicators of executive dysfunction [41]. The four-item version, respectively, assesses self-organisation/problem solving, self-management of time, self-restraint, self-regulation of emotion, and self-motivation [40,41]. While models examining BDEFS-SF sub-scale scores might have revealed group by session interactions, recent findings suggest that total, but not sub-scale, scores account for the majority of variance in BDEFS-SF scores [41]. The mean scores of participants in our sample ranged between 27.14 and 31.64. By contrast, other studies have reported mean scores of 39.30 and 32.98, respectively, in young adult (*M_age_* = 20.00) and adult (*M_age_* = 35.48) samples [41,60]. Our lower average scores might have been reflective of the older age of our participants (*M_age_* = 47.72). Self-reported executive function has been shown to improve with age, with individuals 18–39 years reporting a significantly greater number of deficits than those 40–59 years [61]. The distribution of scores at all timepoints in our sample was also positively skewed, with between 5 and 19% of the sample showing floor effects at any one timepoint. It is therefore likely that 20 items were not sufficient to fully chart variations in executive function in our sample across time. Finally, unlike the BIS, the BDEFS-SF is a more general measure of executive function. As such, it may not be specific enough to capture the component of executive function that DEX treatment targets in the case of obesity. In future studies, it would be interesting to include a measure that taps self-reported impulsivity, such as the BIS, alongside a longer more general measure of executive function.

Regarding response inhibition, modelling showed a main effect of time on SSRT, with scores at Baseline being significantly higher than scores at all other sessions, as well as a main effect of group, with mean SSRT in the control group being significantly greater than the treatment group. Crucially, there was no group by session interaction, suggesting that any changes in SSRT across time were not related to DEX treatment. Differences in performance on the SST between Baseline and all other sessions were likely due to practice effects. Several studies have shown significant differences between SSRT [47,62] across two testing sessions. While studies investigating reliability of the task over a greater number of sessions are limited, a recent paper reported a significant main effect of session on a shorter version of the task undertaken at eight timepoints, with sessions one and two characterised by longer SSRT values than later sessions [63]. The same study reported an ICC of 0.53 [63]. From a psychometric perspective, our results appear to be in keeping with these findings. Collectively, these results suggest that preliminary practice sessions might be a valuable addition to psychopharmacological studies looking to use the SST, particularly when a longitudinal design is employed. Main effects differences between groups in this study likely reflect pre-existing differences between the groups. While our preliminary analyses showed no age, weight, or biological sex differences between the groups, there may have been other differences, such as education or mood, that contributed to this finding. Other studies, however, have reported no association between performance on the SST and education [47] or mood [62]. Pre-existing differences between groups might also be a function of the relatively modest sample size in this study.

Our finding of no change in response inhibition as a function of treatment group accords with results of a previous study that found no improvement in patients being treated with lisdexamfetamine for binge eating disorders [39]. These results are nonetheless somewhat surprising given the literature suggesting impaired response inhibition—assessed using a variety of objective tasks—in a range of eating-related disorders, including obesity, as compared to healthy controls [35,36,37]. Again, this work contributes to a body of evidence suggesting parallels between underling mechanisms in eating-related disorders and ADHD [6]. Given that there were no healthy controls in this study, however, it is difficult to ascertain whether the SSRT values of participants were significantly lower at Baseline than would be expected in the healthy population. Typically, SSRT values are reported in the range of 240–270 ms for healthy control adults [47,64,65]. Participants in this study had similar values (253–283 ms). As such, it is possible that they would not have been characterised by Baseline differences relative to healthy controls. If this was the case, improvements beyond practice effects would have been unlikely. Alternatively, the task might not be sensitive enough to chart response inhibition in the case of obesity. Griffiths and colleagues have proposed the use of food-related response inhibition tasks in future studies, which may increase the sensitivity of the task in eating-related disorders [39].

While a strength of this pilot study was our use of both subjective self-report and objective task-based measures to, respectively, chart executive function broadly and response inhibition specifically, several general limitations are worth highlighting. Sample size was relatively modest with 47 individuals (after criteria-based exclusions) randomised to two group. Similar studies have had larger samples [8,39]. Nonetheless, post hoc power analyses using G*Power 3.1.9.7 [66] determined that this study (α = 0.05; *n* = 47; number of groups two; number of measurements nine) was sufficiently powered to detect medium effects (*f* = 0.25; power = 0.99), though underpowered in the case of small effects (*f* = 0.10; power = 0.49). Unfortunately, the COVID-19 pandemic impacted timely data collection. Rolling lockdowns and associated difficulties during this period meant participants were not always able to attend the laboratory as required. As such, there was a higher-than-expected amount of missing data at each timepoint, which impacted power. Interestingly, a comparable recent study also conducted during the pandemic detailed similar sample size and missing data difficulties. They investigated how cognitive-behavioural therapy impacted executive function and weight loss using an intervention (*n* = 24) versus control (*n* = 26) design [67]. The current study also did not include a healthy control group. This meant we were unable to determine whether baseline executive function or response inhibition in our sample of obese individuals was significantly different from that of healthy controls. Possibly, no group by session interactions were evident in this study because our sample was not characterised by deficits in these areas. Finally, treatment group participants were given DEX, whereas other similar studies investigating executive function and response inhibition have utilised lisdexamfetamine [8,39]. Amphetamines differ in terms of the structure and mechanism of effect [1,5]. While DEX is a single molecule composed entirely of dextroamphetamine, lisdexamfetamine is a prodrug and, as such, it is metabolised by the body into dextroamphetamine and the amino acid lysine [68]. It consequently has a slower onset of action and longer duration of effect than DEX. Moreover, DEX enhances DA release from nerve terminals via transporter-mediated reverse processes, whereas lisdexamfetamine inhibits DA transporters, blocking reuptake into the presynaptic neuron [10,69]. These subtle differences may be impacting cognitive processes in distinct ways.

## 5. Conclusions

In sum, this double-bind randomised controlled pilot study found no evidence that DEX treatment for obesity improved executive function generally or response inhibition specifically. While results might be related to survey-/task-specific limitations, the small sample size, or differences in mechanism of drug action, it is also possible that our sample was not characterised by executive function or response inhibition deficits at Baseline. Although further research is required, our findings call into question parallels between eating-related disorders and ADHD in terms of mid-brain-mediated impulsivity and top-down executive function. Reassuringly, however, our data also suggest that DEX treatment does not render individuals susceptible to SUD.

## Figures and Tables

**Table 1:** Average Barkley Deficits in Executive Functioning Scale—Short Form score for the sample across sessions.

	Baseline (*n* = 47)	Time 1 (*n* = 40)	Time 2 (*n* = 41)	Time 3 (*n* = 41)	Time 4 (*n* = 41)	Time 5 (*n* = 40)	Time 6 (*n* = 37)	Maintenance (*n* = 36)	Follow-up (*n* = 33)
	*M* (*SD*)	*M* (*SD*)	*M* (*SD*)	*M* (*SD*)	*M* (*SD*)	*M* (*SD*)	*M* (*SD*)	*M* (*SD*)	*M* (*SD*)
BDEFS-SF *	27.51 (7.05)	30.15 (7.97)	29.56 (9.19)	28.85 (7.93)	28.22 (7.48)	28.13 (7.31)	27.60 (7.59)	27.14 (7.98)	31.64 (12.51)

* BDEFS-SF = Barkley Deficits in Executive Functioning Scale—Short Form.

**Table 2:** Average performance on Stop-Signal Task variables for the sample (after exclusions) across sessions.

	Baseline (*n* = 40)	Time 1 (*n* = 32)	Time 2 (*n* = 36)	Time 3 (*n* = 31)	Time 4 (*n* = 36)	Time 5 (*n* = 34)	Time 6 (*n* = 40)	Maintenance (*n* = 32)	Follow-up (*n* = 31)
	*M* (*SD*)	*M* (*SD*)	*M* (*SD*)	*M* (*SD*)	*M* (*SD*)	*M* (*SD*)	*M* (*SD*)	*M* (*SD*)	*M* (*SD*)
Go accuracy (%)	98.27 (2.12)	97.95 (6.27)	98.13 (5.50)	98.86 (1.17)	99.06 (1.51)	99.32 (0.76)	97.84 (5.63)	98.81 (1.49)	98.79 (2.29)
Go RT (ms)	552.88 (138.19)	573.95 (151.19)	618.09 (159.76)	620.37 (178.08)	641.98 (160.32)	638.07 (156.97)	612.59 (165.08)	607.86 (178.72)	641.90 (176.92)
Go omissions (%)	0.81 (1.90)	1.47 (5.95)	1.39 (4.70)	0.75 (0.98)	0.69 (1.42)	0.37 (0.68)	1.54 (5.61)	0.60 (1.13)	0.84 (2.02)
Go errors (%)	0.92 (1.15)	0.57 (0.92)	0.49 (1.22)	0.39 (0.81)	0.25 (0.75)	0.29 (0.58)	0.62 (1.45)	0.60 (1.19)	0.37 (0.88)
Stop accuracy (%)	49.05 (4.82)	49.06 (6.15)	48.37 (4.83)	48.66 (3.59)	48.92 (5.14)	48.72 (5.73)	49.23 (6.25)	49.73 (5.00)	48.18 (5.13)
Failed stop RT (ms)	483.59 (105.67)	511.68 (130.47)	553.21 (139.29)	556.12 (152.27)	578.76 (148.62)	568.38 (133.40)	544.40 (139.21)	546.94 (151.76)	573.02 (152.70)
SSD * (ms)	268.37 (147.54)	308.13 (163.84)	355.37 (168.18)	358.26 (183.08)	378.68 (165.88)	384.40 (166.90)	358.27 (167.80)	343.30 (189.47)	386.25 (189.78)
SSRT ** (ms)	283.21 (33.70)	265.24 (35.00)	262.33 (39.84)	261.87 (31.29)	262.93 (31.18)	253.21 (34.90)	253.67 (33.27)	263.78 (33.34)	255.30 (35.65)

* SDD = stop-signal delay; ** SSRT = stop-signal reaction time.

**Table 3:** Minutes between treatment (dexamphetamine or placebo) and undertaking Stop-Signal Task.

	Minutes
	*M* (*SD*)
Baseline	-
Time 1	169.14 (99.34)
Time 2	165.56 (92.50)
Time 3	148.07 (82.81)
Time 4	190.00 (125.44)
Time 5	150.94 (68.97)
Time 6	154.50 (83.70)
Maintenance	167.81 (78.34)
Follow-up	-

## Data Availability

Data are currently unavailable due to ethical restrictions; namely, data collection continues in the overarching weight loss/obstructive sleep apnoea study.

## Supplementary Materials

The following supporting information can be downloaded at the following website: https://www.mdpi.com/article/10.3390/brainsci14121274/s1, Supplementary Results and Figure S1: Stop-Signal Task go reaction time across sessions as a function of treatment group.
