# Supplementary material for: The Impact of Dexamphetamine Treatment for Obesity on Executive Function: A Double-Blind Randomised Controlled Pilot Study
Source: Brain Sci. 2024 Dec 18;14(12):1274. doi: 10.3390/brainsci14121274 (PMC11674214; doi:10.3390/brainsci14121274)
Supplement: Supplementary file 1 [file brainsci-14-01274-s001.zip › brainsci-3366553-supplementary.pdf]

The impact of dexamphetamine treatment for obesity on executive function: A double-blind randomised controlled pilot study

Supplementary Materials

Supplementary Results

For the go RT model, fixed effects omnibus tests revealed no effect of condition on go RT, $F(1, 43.94) = 0.04$, $p = .843$. There was an effect of session, $F(8, 251.58) = 5.02$, $p < .001$, but no effect by session interaction, $F(8, 251.58) = 0.66$, $p = .730$. Examining fixed effects parameter estimates using helmert coding showed that mean Baseline go RT was significantly faster than the mean of all subsequent sessions, beta = -72.30 (*SE* = 14.62), $t(251.61) = -4.95$, 95%CI [-100.96, -43.65], $p < .001$. Similarly, Time 1 go RT was significantly faster than that of all subsequent sessions, beta = -59.23 (*SE* = 16.38), $t(252.26) = -3.62$, 95%CI [-91.34, -27.13], $p < .001$. No other fixed effects parameter estimates were significant. R^2 (marginal) was 0.04; R^2 (conditional) was 0.75. The ICC value was 0.74. Figure S1 shows go RT values across sessions as a function of condition.

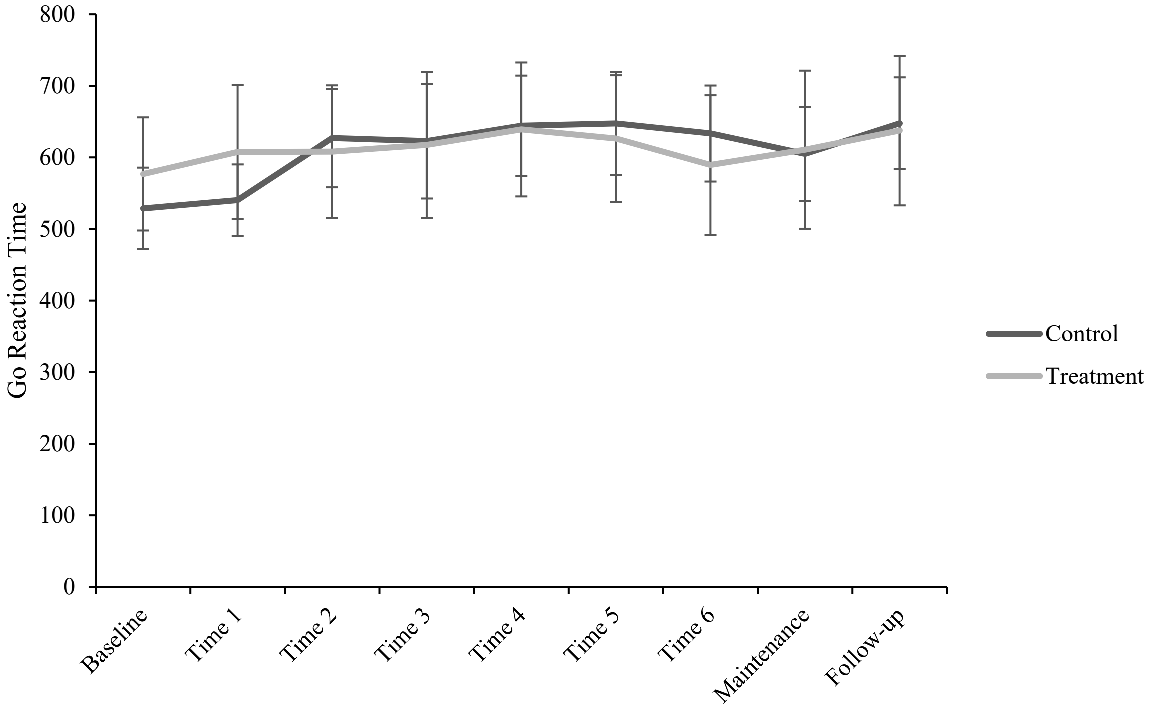

Figure S1. Stop-Signal Task go reaction time across sessions as a function of treatment group.
